# Stage IV renal cell carcinoma achieves pathologic complete response after two ipilimumab plus nivolumab courses despite severe immune-related adverse events: a case report

**DOI:** 10.1186/s40780-024-00348-8

**Published:** 2024-05-31

**Authors:** Ryo Takada, Miki Fujiwara, Masatoshi Maki, Naoyuki Nomura, Shintaro Kono, Akira Fujita, Hiroshi Masumoto, Yoko Takahashi, Yasuhisa Hasegawa, Koji Tamura

**Affiliations:** 1 Department of Hospital Pharmacy, National Hospital Organization Fukuyama Medical Center, 4-14-17 Okinogami-cho, Fukuyama City, Hiroshima 720-8520 Japan; 2 Department of Urology, National Hospital Organization Fukuyama Medical Center, Fukuyama, Japan

**Keywords:** Renal cell carcinoma, Immune checkpoint inhibitors, Ipilimumab, Nivolumab, irAEs

## Abstract

**Background:**

Ipilimumab (Ipi) plus nivolumab (Nivo) is the recommended first-line treatment for renal cell carcinoma (RCC). This report describes a case where pancreatic metastases disappeared after only two courses of Ipi + Nivo therapy. The primary tumor was cured by surgery, and a pathological Complete Response (pCR) was observed despite multiple serious immune-related Adverse Events (irAEs).

**Case presentation:**

A 54-year-old woman with RCC and pancreatic metastasis at stage IV, diagnosed with intermediate risk according to the International Metastatic RCC Database Consortium classification, underwent initiation of Ipi + Nivo therapy. On day 26, she developed hyperthyroidism accompanied by tachycardia, leading to the commencement of metoprolol tartrate treatment. Following the resolution of tachycardia, a second course of Ipi + Nivo therapy was administered on day 50. By day 70, the patient exhibited Grade 3 hepatic dysfunction, followed by the onset of hypothyroidism on day 75, necessitating treatment with steroids and levothyroxine. After positive treatment, a Grade 3 skin disorder emerged on day 87 while tapering steroids, prompting treatment with methylprednisolone (mPSL) pulse therapy. The skin disorder responded to steroids, allowing for tapering. However, on day 113, a recurrence of Grade 3 skin disorder occurred, necessitating another mPSL pulse. The patient responded well to treatment, exhibiting improvement in her condition. On day 131, she presented at the hospital with complaints of respiratory distress, prompting a Computed Tomography (CT) scan that revealed interstitial pneumonia. By day 272, subsequent CT imaging showed the disappearance of pancreatic metastasis and shrinkage of the primary tumor. On day 294, she underwent a laparoscopic left nephrectomy. Pathological analysis confirmed a pCR in the primary tumor, indicating successful eradication of RCC through surgical intervention.

**Conclusions:**

This case report presents a scenario where multiple severe irAEs appeared in a patient, yet metastases disappeared after only two courses of Ipi + Nivo therapy. The patient was ultimately cured by surgery and achieved a pCR. This case highlights that despite the occurrence of severe irAEs during RCC treatment with Ipi + Nivo therapy, they can be managed appropriately to maximize the therapeutic effects of checkpoint inhibitors.

## Background

The advent of immune checkpoint inhibitors (ICIs) has transformed the treatment of metastatic renal cell carcinoma (RCC). In Japan, combination therapy with ipilimumab (Ipi), an anti-cytotoxic T-lymphocyte-associated antigen 4 (CTLA-4) antibody, and nivolumab (Nivo), an anti-programmed cell death-1 (PD-1) antibody, was approved in 2018 for the treatment of pallidum clear cell metastatic renal cancer. Currently, it is recommended as a first-line treatment for intermediate to high-risk patients according to the International Metastatic Renal Cell Carcinoma Database Consortium (IMDC) classification [1, 2]. However, immune-related adverse events (irAEs) have been reported as typical side effects of Ipi + Nivo therapy [3], which can be challenging to manage in severe cases. Nevertheless, reports [4, 5] indicate that the occurrence of irAEs affects prognosis, emphasizing the importance of their control.

Here, we report the case of a patient with stage IV renal cell carcinoma who achieved radical cure and pathologic complete response (pCR) to surgery after only two courses of Ipi + Nivo therapy despite experiencing multiple serious irAEs. Our findings underscore the importance of effectively managing irAEs in patients undergoing combination therapy with ipilimumab and nivolumab for metastatic renal cell carcinoma, ultimately improving treatment outcomes and patient care.

## Case presentation

A 54-year-old woman with no significant medical history or medications and an Eastern Cooperative Oncology Group Performance status of 0 presented with gross hematuria 40 days before chemotherapy. She sought medical attention from her previous physician, who identified a left renal tumor. On 38 days before chemotherapy, a blood clot led to left urinary tract obstruction and pyelonephritis, necessitating treatment. Subsequently, the patient was referred to our urology department on 30 days before chemotherapy for further treatment of the left renal tumor. Biopsies of the renal and pancreatic tumors observed on Computed Tomography (CT) were performed 18 days before chemotherapy, revealing clear cell carcinoma in the renal tumor and metastatic carcinoma in the pancreatic tumor. The pathological findings of the pancreatic tumor were consistent with renal cancer metastasis, and the clinical diagnosis was stage IV renal cell carcinoma (cT3aN0 or 1M1 OTH) with intermediate risk according to IMDC criteria, and the plan was to start Ipi + Nivo therapy. The patient received Ipi (69.5 mg/body; 1 mg/kg) + nivolumab (240 mg) on day 1 in the hospital, completed the treatment without any adverse effects, and was discharged after several days of follow-up.

**Fig. 1:**
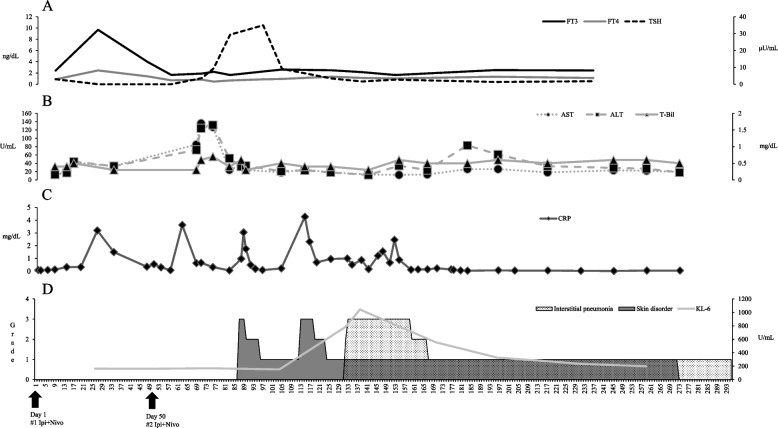
Overall summary of this case. **A** Thyroid dysfunction. **B** Impairment of liver function. **C** Serum CRP level. **D** Pulmonary and skin disorders

Upon admission to the hospital on day 26, she reported experiencing palpitations, and her blood sample revealed Thyroid Stimulating Hormone (TSH) levels of 0.024 μU/mL, Free Triiodothyronine (FT3) levels of 9.68 ng/dL, Free Thyroxin (FT4) levels of 2.46 ng/dL, indicating hyperthyroidism. Consequently, the patient was initiated on metoprolol tartrate 60 mg/day (Fig. 1A). During a follow-up visit on day 47, although TSH remained low at 0.024 μU/mL, FT3 levels had decreased to 3.97 ng/dL and FT4 levels to 1.4 ng/dL, showing a trend towards recovery. Additionally, her tachycardia improved. Consequently, the dose of metoprolol tartrate was reduced to 40 mg/day, and treatment was discontinued after 1 week. With the improvement in thyroid function, a second dose of Ipi+Nivo was administered on Day 50 at the same dose as the first. On day 68, blood tests revealed elevated levels of aspartate aminotransferase (AST) at 85 U/mL and alanine aminotransferase (ALT) at 72 U/mL, indicating Grade 2 elevation. Treatment with ursodeoxycholic acid (UDCA), 200 mg/day, was initiated. However, on day 70, AST levels increased to 136 U/mL and ALT to 124 U/mL, reaching Grade 3 hepatic dysfunction. Consequently, the dosage of UDCA was increased to 600 mg/day. Owing to the patient’s persistent Grade 3 liver dysfunction (AST=127 U/mL and ALT=132 U/Ml), on day 75, and with a poor response to treatment, prednisolone (PSL) 20 mg/day was initiated on suspicion of irAE liver injury. At this point, while FT3 and FT4 levels were within the lower limits of normal, TSH levels were notably elevated at 9.415 μU/mL, necessitating the addition of Levothyroxine (LT4) at a dosage of 25 μg/day. Subsequent improvement in liver function was observed, with AST=24 U/mL and ALT=52 U/mL on day 82, leading to a reduction in PSL dosage to 15 mg/day (Fig. 1B). During the follow-up period, AST and ALT levels normalized, but LT4 dosage was increased to 50 μg/mL owing to further elevation in TSH to 29.391 μU/mL on day 82 (Fig. 1A).

**Fig. 2:**
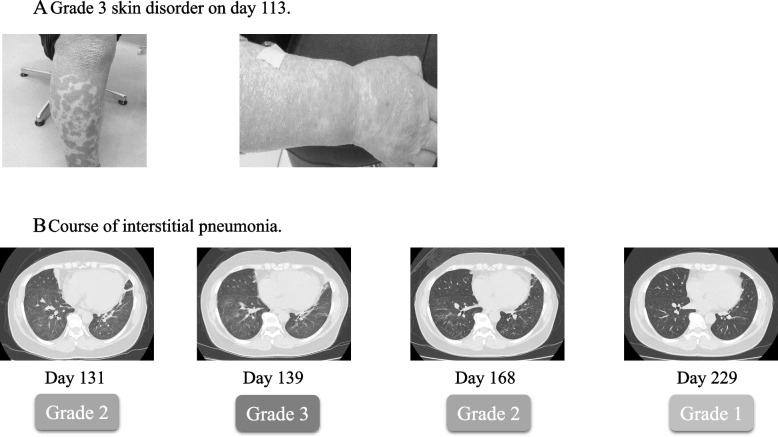
Images of skin disorders and CT images of interstitial pneumonia. **A** Grade 3 skin disorder on day 113. **B** Course of interstitial pneumonia

However, on day 87, the patient underwent urgent examination owing to generalized redness and pruritus, resulting in a diagnosis of Grade 3 skin disorder. Intravenous methylprednisolone (mPSL) was initiated at a dosage of 125 mg/body (Fig. 1D). By day 90, with the administration of steroids, the patient’s condition improved to Grade 2 equivalent. Subsequently, treatment was transitioned to 1.5 mg of betamethasone based on the dermatologist’s judgment. By day 96, the patient further improved to a Grade 1 equivalent, prompting a reduction in the betamethasone dosage to 1.0 mg. In addition, a blood sample collected on day 96 revealed elevated TSH levels, peaking at 34.918 μg/mL. However, on day 113, upon tapering off the steroid, Grade 3 skin lesions reappeared (Fig. 2A), necessitating urgent hospitalization and the resumption of 125 mg mPSL (Fig. 1D). By day 119, the patient had improved to a Grade 2 equivalent, prompting a transition to PSL 60 mg and the initiation of tapered therapy as an outpatient. Nonetheless, the patient presented to the hospital on day 131 owing to dyspnea. CT revealed Grade 2 interstitial pneumonia (Fig. 2B), leading to urgent hospitalization and the resumption of 125 mg mPSL. Upon admission, the patient was evaluated for Grade 3 interstitial pneumonia owing to the need for oxygenation. However, she responded positively to steroids and was able to reduce her dosage to PSL 60 mg gradually. However, on day 139, his respiratory condition worsened again, and CT imaging revealed worsening interstitial pneumonia. A respiratory physician diagnosed this as a hypersensitivity pneumonitis (HP) pattern (Fig. 2B).

**Fig. 3:**
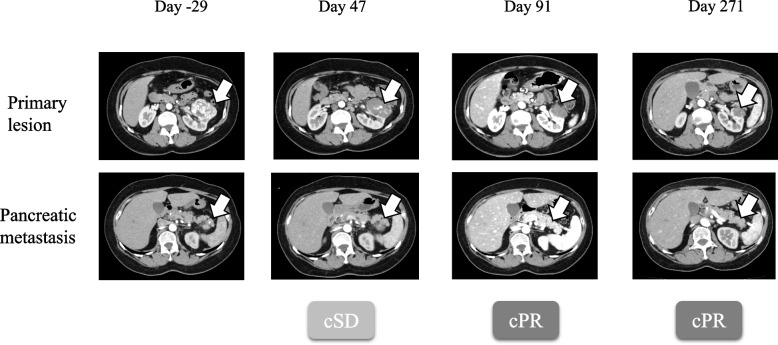
Therapeutic effects

Consequently, she commenced a 3-day pulse therapy with 1000 mg of mPSL. Furthermore, because pulse therapy with 1000 mg mPSL alone failed to sufficiently improve oxygenation, on day 148, she began pulse therapy with 125 mg of mPSL in addition to 500 mg/body cyclophosphamide (CPA). Following the initiation of CPA pulse therapy, oxygenation improved, enabling the tapering off of steroids, with mPSL reduced to 80 mg on day 154 and PSL to 60 mg on day 159, eventually transitioning to oral PSL and subsequent tapering. By day 272, CPA pulses had been administered nine times, improving interstitial pneumonia to Grade 1 equivalent, and PSL was tapered to 7.5 mg. On day 271, CT scans revealed that the primary tumor remained diminished, the pancreatic metastasis had disappeared (Fig. 3), the skin lesion had resolved, and interstitial pneumonia had improved to a mild Grade 1 (Fig. 1D). Consequently, a laparoscopic left nephrectomy was performed on day 294. Intraoperative findings showed no invasion of the pancreas, leading to the decision not to perform a pancreatectomy. Histological evaluation of the resected left kidney showed a pCR, and the patient was successfully treated with curative surgery. During the outpatient visit on day 304, the PSL was reduced to 5 mg. An attempt was made to change the PSL to hydrocortisone; However, the patient developed bronchiolitis obliterans, prompting an increase in the PSL back to 30 mg. The PSL was not discontinued entirely in this case. To date, no evidence of recurrence has been reported for RCC.

## Discussion and conclusions

In this case, we encountered a scenario where, despite the emergence of severe and diverse irAEs, the pancreatic metastasis disappeared after only two courses of Ipi + Nivo therapy, and the primary tumor was eradicated through surgery, achieving a pCR. Since this patient had no specific medical history or medication history, no prior treatments or medication history could affect the efficacy of Ipi + Nivo. In addition, the only medications taken regularly before surgery were lansoprazole, LT4, sulfamethoxazole/trimethoprim fixed-dose combination, and UDCA, which are unlikely to affect the effectiveness of Ipi + Nivo. Although the outcomes of RCC treated with Ipi + Nivo have been reported [6–9], to our knowledge, there is no prior report of a case where the primary tumor shrank, pancreatic metastases disappeared, and various severe irAEs developed after only two courses, as seen in our case, followed by cure through surgical intervention.

Tumor cells have many mechanisms by which they can evade surveillance by the immune system. Immune checkpoints, such as CTLA-4 and PD-1, have been implicated in tumor evasion [10]. Blockade of the PD-1 pathway triggers an adaptive memory immune response that resets the equilibrium between the tumor and the host immune response [11, 12]. Hence, it has the potential to sustain an anti-tumor response after treatment cessation, which may also translate to ongoing immune-related toxicities [13]. In this case, Ipi + Nivo was last administered on day 50, with continued irAEs and anti-tumor response. To identify predictors of response and survival, an investigation into the tumor and immune microenvironment is necessary. However, preoperative pathology revealed only immunostaining of renal cell carcinoma, and postoperative pathology indicated tumor cell disappearance, making it impossible to assess the tumor microenvironment definitively. Therefore, definitive conclusions about the microenvironment around the tumor are not possible. Clear cell RCC, the most prevalent subtype of RCC, is considered an immunogenic disease [14, 15]. Emerging evidence indicates that Clear RCC typically exists in an immunosuppressive state characterized by the high infiltration of regulatory T cells and myeloid cells with suppressive activity [16, 17]. Additionally, studies have reported that tumor microenvironments where ICI are effective have significantly more effector T cells and fewer inhibitory T cells [18], indicating that the microenvironment, in this case, was conducive to ICI effectiveness.

Alternatively, recent clinical reports have highlighted commonalities between ICI and ICI combination therapies. One such commonality is the occurrence of irAEs, while another is C-reactive protein (CRP) control status.

Regarding the occurrence of irAEs, studies indicate that prognosis improves when irAEs manifest, even in renal cell carcinoma during ICI treatment [4], and when multiple types of irAEs occur in other cancer types [5]. Additionally, specific types of irAEs, such as vitiligo and skin lesions, have been associated with enhanced overall survival [4, 5]. Although the occurrence of interstitial pneumonia has been reported to have no prognostic value [5], the risk of pneumonia was reported to be 3.68 times higher in the Nivo group compared to the Ipi alone group for all grades [19]. Furthermore, Grade 3 or higher pneumonia was significantly more common in the Ipi + Nivo group compared to the Nivo alone group [20], and interstitial pneumonia can be fatal in some cases, necessitating caution.

In addition, studies have indicated that prognosis is more favorable when an irAE emerges early in lung cancer within 42 days of treatment initiation [21], with the first irAE appearing within this timeframe. In our case, both skin disorder and interstitial pneumonia manifested. However, both steroids and pulse therapy with CPA, an effective immunosuppressive agent for interstitial pneumonia [22], were effective, especially in managing interstitial pneumonia, which we believe influenced the prognosis.

**Fig. 4:**
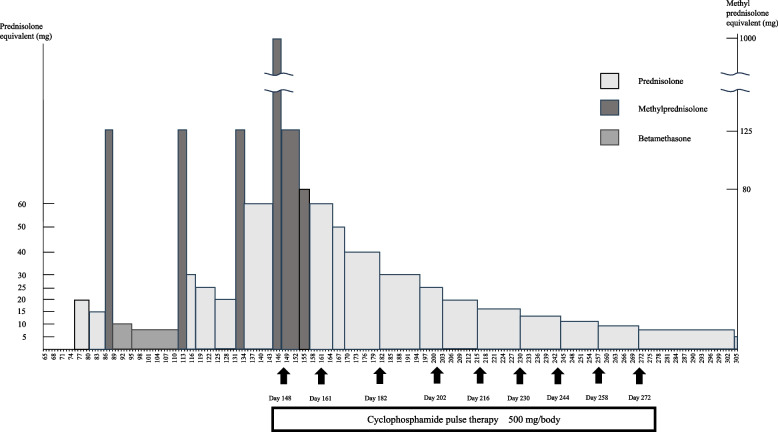
Course of immunosuppressive drugs, including steroids in this case

The trends in CRP level in this case are shown in Fig. 1C. CRP levels were low before treatment initiation, increased with the onset of each irAE appeared, and decreased upon irAE control. In this case, the elevation of CRP was not accompanied by an increase in white blood cell count or neutrophil count, potentially indicating inflammation induced by the appearance of irAE. A CRP level of ≥ 1 mg/dL before commencing nivolumab treatment for RCC is considered a poor prognostic indicator [23], with studies suggesting that CRP control during ICI treatment for RCC contributes to overall survival [24]. A meta-analysis showed that maintaining CRP levels below 10 mg/dL during ICI treatment impacts prognosis [25]. In our case, although CRP levels were elevated during irAE occurences, they did not surpass 10 mg/dL. The dose and duration of immunosuppressive drugs, including steroids, used in this case are also shown in Fig. 4. Nonsteroidal Anti-Inflammatory Drugs were not administered to this patient during the study period. As shown in Fig. 4, although steroids were initiated on day 75, the CRP level did not rise above 10 mg/dL before or after the start of steroids, indicating that immunosuppressive drugs likely did not affect CRP suppression. Therefore, the CRP level was suppressed below 10 mg/dL without being affected by other factors, indicating that this may have affected efficacy as in this case.

Regarding interstitial pneumonia, the HP pattern was diagnosed and treated in this case. Various interstitial pneumonia patterns are associated with Nivo, and the HP pattern has a relatively good prognosis and is effective with steroid therapy [26, 27]. In this case, the patient was treated with steroids and CPA pulses before the interstitial pneumonia progressed to fibrosis, potentially preventing its worsening.

Referring to Fig. 4, it is indicated that this patient switched from mPSL to betamethasone at the time of the first skin disorder. However, the decrease in steroid equivalent was significant and may have influenced the appearance of the second skin disorder. After the diagnosis of grade 3 interstitial pneumonia, the CPA pulse could have been administered sooner, but was delayed until day 148. This delay was due to a successful pulse with 125 mg mPSL, which allowed the dose to be reduced to 60 mg PSL. Alternatively, although being on steroids for approximately 220 days, the patient did not experience adverse events such as steroid-induced peptic ulcer, pneumocystis pneumonia, or steroid-induced diabetes mellitus. This may be attributed to the pharmacist’s suggestion and the patient’s acceptance of a prescription for a 30 mg dose of lansoprazole for peptic ulcer from day 75 and a fixed dose of sulfamethoxazole/trimethoprim for pneumocystis pneumonia from day 132 as prophylactic administration. In addition, in our hospital, when employing ICI, the pharmacy department prepares a test set for baseline confirmation of irAE and blood collection items for ongoing ICI administration. In this case, the pharmacist recommended the test set to the physician, facilitating baseline confirmation before ICI administration, and subsequently for continued ICI use, enabling early response to irAE, such as thyroid dysfunction and liver dysfunction, based on test values. It is commendable that this has made it possible. However, prophylactic administration of bisphosphonates for osteoporosis was not met. Although there was no pre-existing fracture in this case, the patient’s age in the 50 s and long-term use of steroids at a PSL equivalent of 5 mg/day or more suggest that prophylactic administration of bisphosphonates for osteoporosis was necessary according to guidelines [28]. In retrospect, fortunately, no fractures occurred, and steroid-induced osteoporosis was avoided. However, we regret that we were distracted by the appearance of irAE and failed to propose prophylactic administration against steroid-induced osteoporosis.

Study limitations exist in this case report. The first is that no bronchoscopy or other examination was performed when interstitial pneumonia appeared, and there are no pathological findings regarding interstitial pneumonia. However, the respiratory medicine department diagnosed the HP pattern on CT findings. Since the respiratory medicine department took the lead in providing appropriate treatment for interstitial pneumonia, the pneumonia did not turn out to be fatal. Another limitation is the lack of immunocyte staining of pathology specimens. Some previous studies looking into pathologic features of responders to immunotherapy suggested that programmed cell death-ligand 1(PD-L1) expression, tumor infiltration with CD8 + T lymphocytes and tumor mutational burden are predictive biomarkers in ICI therapy [29, 30]. In this case, the tumor was pCR, and there was no residual tumor; therefore, it was impossible to determine the PD-L1 expression status of the tumor or other information by pathology.

In conclusion, we presented a case wherein a single patient experienced multiple severe irAEs yet achieved the disappearance of pancreatic metastases, primary tumor shrinkage, radical cure through surgical intervention, and pCR after only two courses of Ipi + Nivo therapy. Healthcare providers should be aware that even in the presence of severe irAEs during ICI treatment for RCC, appropriate management can optimize the therapeutic outcomes of ICI. Further studies are needed to validate the results of this report and explore the potential benefits and limitations of Ipi + Nivo therapy.

## Data Availability

The datasets used and analyzed during the current study are available from the corresponding author upon reasonable request.

## Abbreviations

ICI: Immune Checkpoint inhibitor
RCC: Renal Cell Carcinoma
Ipi: Ipilimumab
CTLA-4: Cytotoxic T-lymphocyte-associated antigen 4
Nivo: Nivolumab
PD-1: Programmed cell death-1
IMDC: International Metastatic Renal Cell Carcinoma Database Consortium
irAEs: Immune-related Adverse Events
pCR: Pathologic complete response
TSH: Thyroid Stimulating Hormone
FT3: Free Triiodothyronine
FT4: Free Thyroxin
AST: Aspartate aminotransferase
ALT: Alanine aminotransferase
UDCA: Ursodeoxycholic acid
PSL: Prednisolone
LT4: Levothyroxine
mPSL: Methylprednisolone
CT: Computed Tomography
HP: Hypersensitivity pneumonitis
CPA: Cyclophosphamide
CRP: C-Reactive Protein
PD-L1: Programmed cell death-ligand 1
